# Analysis of immune cell components and immune-related gene expression profiles in peripheral blood of patients with type 1 diabetes mellitus

**DOI:** 10.1186/s12967-021-02991-3

**Published:** 2021-07-26

**Authors:** Jian Lin, Yuanhua Lu, Bizhou Wang, Ping Jiao, Jie Ma

**Affiliations:** 1 Department of Regenerative Medicine, School of Pharmaceutical Sciences, Jilin University, 1266 Fujin Road, Changchun, Jilin 130021 P.R. China; 2 Department of Prosthodontics, Hospital of Stomatology, Jilin University, Changchun, Jilin 130021 P.R. China

**Keywords:** Type 1 diabetes mellitus, Peripheral blood, Immune cells, Gene expression profiles

## Abstract

**Background:**

Type 1 diabetes mellitus (T1DM) is a chronic autoimmune disease caused by severe loss of pancreatic β cells. Immune cells are key mediators of β cell destruction. This study attempted to investigate the role of immune cells and immune-related genes in the occurrence and development of T1DM.

**Methods:**

The raw gene expression profile of the samples from 12 T1DM patients and 10 normal controls was obtained from Gene Expression Omnibus (GEO) database. Differentially expressed genes (DEGs) were identified by Limma package in R. The least absolute shrinkage and selection operator (LASSO)—support vector machines (SVM) were used to screen the hub genes. CIBERSORT algorithm was used to identify the different immune cells in distribution between T1DM and normal samples. Correlation of the hub genes and immune cells was analyzed by Spearman, and gene-GO-BP and gene-pathway interaction networks were constructed by Cytoscape plug-in ClueGO. Receiver operating characteristic (ROC) curves were used to assess diagnostic value of genes in T1DM.

**Results:**

The 50 immune-related DEGs were obtained between the T1DM and normal samples. Then, the 50 immune-related DEGs were further screened to obtain the 5 hub genes. CIBERSORT analysis revealed that the distribution of plasma cells, resting mast cells, resting NK cells and neutrophils had significant difference between T1DM and normal samples. Natural cytotoxicity triggering receptor 3 (*NCR3*) was significantly related to the activated NK cells, M0 macrophages, monocytes, resting NK cells, and resting memory CD4^+^ T cells. Moreover, tumor necrosis factor (*TNF*) was significantly associated with naive B cell and naive CD4^+^ T cell. *NCR3* [Area under curve (AUC) = 0.918] possessed a higher accuracy than *TNF* (AUC = 0.763) in diagnosis of T1DM.

**Conclusions:**

The immune-related genes (*NCR3* and *TNF*) and immune cells (NK cells) may play a vital regulatory role in the occurrence and development of T1DM, which possibly provide new ideas and potential targets for the immunotherapy of diabetes mellitus (DM).

**Supplementary Information:**

The online version contains supplementary material available at 10.1186/s12967-021-02991-3.

## Background

According to the latest statistics from the International Diabetes Federation (IDF) (https://www.idf.org/), the global prevalence of diabetes is about 9.3% (463 million people) in 2019, and it is forecasted to rise to 10.2% (578 million people) by 2030 and to 10.9% (700 million people) by 2045. Type 1 diabetes mellitus (T1DM) caused by autoimmune reaction is a major subtype of diabetes, and is mostly prevalent in adolescents and childhood [1]. Compared with type 2 diabetes mellitus (T2DM), T1DM has higher morbidity, mortality and health care cost [2, 3]. It is widely accepted that T1DM is the result of the interaction of both genetic and environmental factors, but its exact molecular mechanism is still unclear. Over the past 30 years, insulin therapy, immunotherapy and some potential therapies such as cell therapy are the main treatments for diabetes. However, these treatments usually have some problems, for instance, insulin therapy can cause a series of complications, while immunotherapy may impact acquired immunity and the efficacy is short-term [4–6].

To understand the mechanisms lying in the pathogenesis of T1DM, a wide range of studies have been carried out and found that immune responses play an important role in T1DM, which need the coordianated efforts of multiple immune related genes and various immune cells [7–11]. For instance, the onset phase of T1DM is characterized by the perturbations of NK cells. Patients with long-standing T1DM showed reduced NK cell activity due to decreased mRNA expression of the cell surface markers NKp30/p46 [also known as natural cytotoxicity triggering receptor 3/1 (NCR3/1)], as well as IFN-γ and perforin [12]. Th1 cells and regulatory T cells (Tregs), two hypotypes of CD4^+^ T cells, have also been reported to promote the pathogenesis of T1DM by destroying β cells via secreting cytokines like IL-1 [13].

However, the roles of immune related genes and immune cells, as well as the associations between them in T1DM have not been fully investigated. Thus, the current study aims to analyze the immune cell components, immune related genes and their correlations in peripheral blood of T1DM patients via bioinformatic strategies. Our findings will provide novel insights into diagnosis and improve our knowledge of the immunotherapies for T1DM patients.

## Materials and methods

### Dataset acquisition

The raw gene expression profile dataset (GSE55098) was obtained from the National Center of Biotechnology Information (NCBI) Gene Expression Omnibus (GEO) database (http://www.ncbi.nlm.nih.gov/geo/). The samples for this data were the peripheral blood mononuclear cells (PBMCs). The dataset based on GPL570 platform included 12 T1DM patients and 10 normal controls. The 1639 immune-related genes were downloaded from the ImmPort database (Immunology Database and Analysis Portal database, https://www.immport.org/shared/home).

### Identification of immune-related DEGs

The dataset between T1DM patients and normal controls was identified using Limma package in R. The *P* < 0.05 and |Log_2_ fold change (FC)|≥ 0.585 were set as the cut-off for the DEGs. The DEGs were exhibited by the volcano plot and the heatmap. The DEGs were overlapped with the 1639 immune-related genes, getting 50 immune-related DEGs.

### Analyses of the GO and KEGG

The GO enrichment and the KEGG pathway analyses were conducted using the immune-related DEGs respectively by GOseq package and hypergeometric test in R, which was used to explore the potential biological processes (BP), cellular components (CC), molecular functions (MF) and identified significantly relevant signal pathways of the immune-related DEGs. The histogram and bubble chart were plotted by the ggplot2 package (version 1.0.2) in R.

### Analyses of LASSO and SVM

By constructing a penalty function, LASSO can compress variable coefficients and make some regression coefficients to become 0, thereby achieving the purpose of variable selection [14]. To screen the gene signatures, the 50 immune-related DEGs were performed the LASSO regression analysis in the glmnet package in R. SVM is supervised machine learning techniques widely used in pattern recognition and classification problems, which have been used in medical applications to predict whether a new gene falls into one category or the other, thereby classifying the genes [15]. The SVM was also used to screen the gene signatures. The overlapping genes after LASSO and SVM analyses were used as the hub genes.

### CIBERSORT analysis

CIBERSORT algorithm could quantify the abundance of specific cell types [16]. To compare the difference between T1DM samples and normal samples in immune cells, the CIBERSORT analysis (https://cibersort.stanford.edu) was used to estimate the percentage of LM22 (22 immune cell types) in each sample. Moreover, the fraction of 22 immune cells was compared between T1DM samples and normal samples and the violin plot was drawn by vioplot package in R.

### Enriched GO-BP and KEGG network by 50 immune-related DEGs

Correlation of the hub genes and immune cells was analyzed by Spearman and visualized by heatmap in T1DM and normal samples. To systematically explore potential functions between the hub genes, 50 immune-related DEGs were imported into the Cytoscape software v3.7.2 (https://cytoscape.org/) to construct the genes and pathways interaction network by ClueGO plug-in. ClueGO was used to decipher the functionally grouped GO and pathway annotation networks to understand their implication in three different classifications (BP, MF and CC), in addition to the KEGG signaling pathway. The relationship between the terms was calculated using κ statistics and the ClueGO network was built based on the similarity of their related genes. In the present study, the enrichment analysis of gene-BP and gene-pathway was statistically validated using the ClueGO plug-in. BPs/signaling pathways were functionally split into several groups with κ score ≥ 0.4. In network, the node represented a BP/pathway, and the edge between two nodes indicated that the two BPs/pathways shared common genes.

### Statistical analysis

The WilcoxTest were used to compare the fraction of the immune cells between T1DM samples and normal samples in CIBERSORT analysis. ROC curves were used to assess the diagnostic value of genes in T1DM,and the higher the AUC value is, the stronger the diagnostic value will be. The *P* < 0.05 was considerable as the statistical significance.

## Results

### Screening of DEGs between the T1DM samples and normal samples

**Fig. 1:**
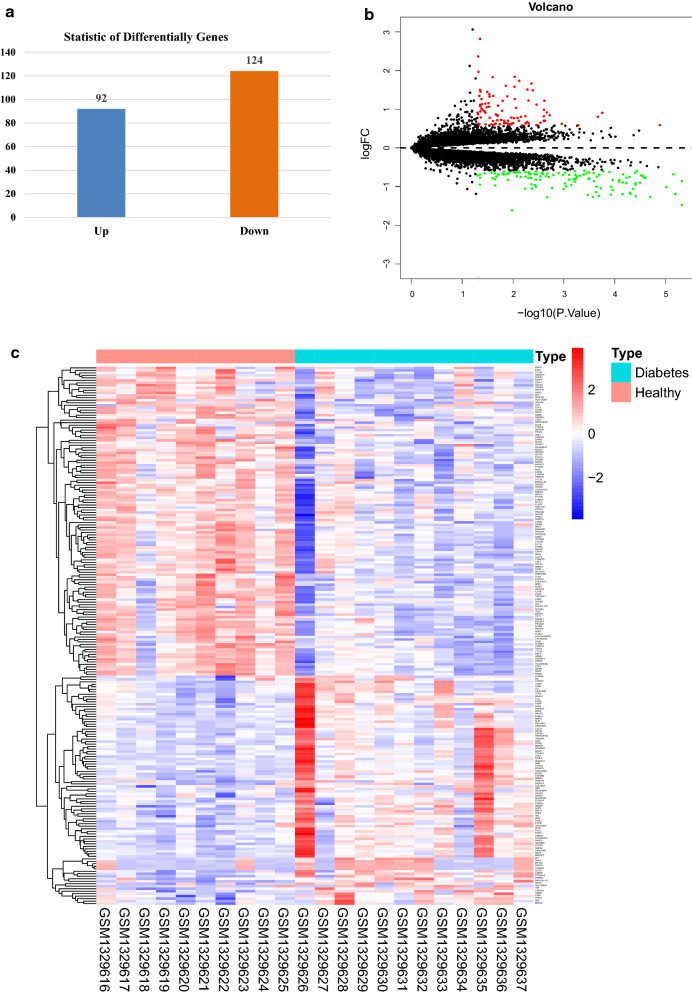
The amount of 216 DEGs between the T1DM samples and normal samples. **a** Histogram; **b** volcano plot; **c** heatmap

The 216 DEGs between the T1DM samples and normal samples were screened out (Fig. 1a), including 92 up-regulated genes (Additional file 1: Table S1) and 124 down-regulated genes (Additional file 2: Table S2). The distribution of the DEGs was displayed by the volcano plot (Fig. 1b), and the expressions of the DEGs in each sample were shown in Fig. 1c.

### The raw identification and functional enrichment analysis of the immune-related DEGs

**Fig. 2:**
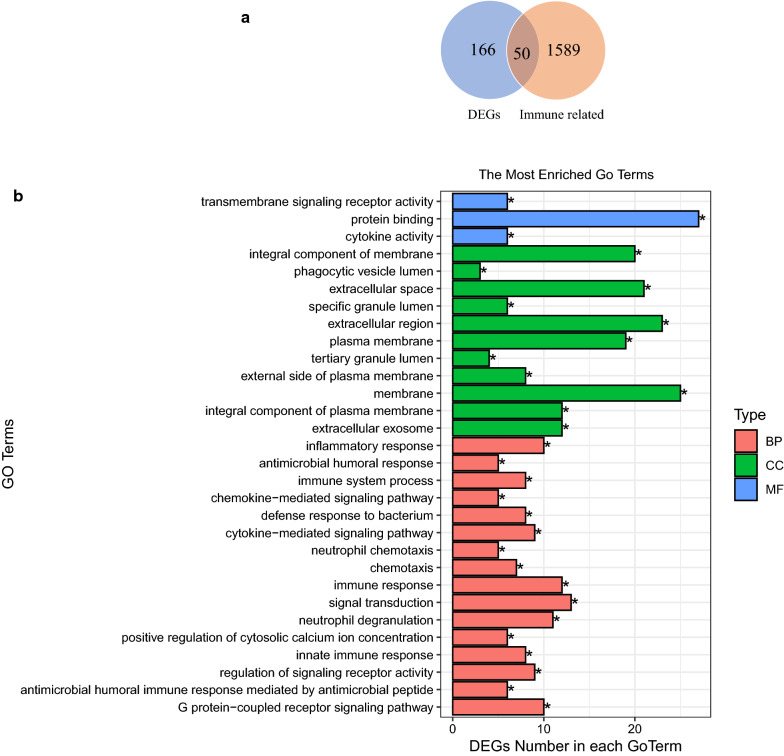

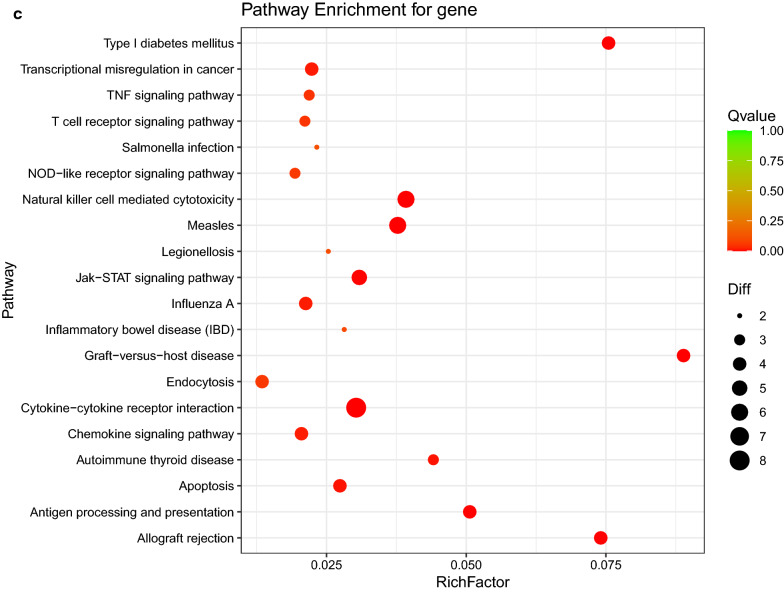
Raw identification and functional enrichment analysis of the immune-related DEGs. **a** Venn diagram of overlapping genes; **b** GO terms; **c** KEGG pathway

The 1639 immune-related genes were overlapped with the DEGs using the Venn diagram, getting 50 immune-related DEGs (Fig. 2a) that were used for subsequent analysis.

To further investigate the functions of the immune-related DEGs, the GO and KEGG analyses were performed. The GO terms with the top 5 of gene enrichment were protein binding, membrane, extracellular region, extracellular space, and integral component of membrane (Fig. 2b and Additional file 3: Table S3), suggesting that the immune-related DEGs may be involved in the biological activities of the cell membrane. Meanwhile, the analysis of KEGG pathway indicated that these genes were mainly enriched in Type 1 diabetes mellitus pathway (Fig. 2c and Additional file 4: Table S4), further verifying that the immune-related DEGs played a vital role in the occurrence and development of T1DM.

### Identification of the optimal immune-related biomarkers

**Fig. 3:**
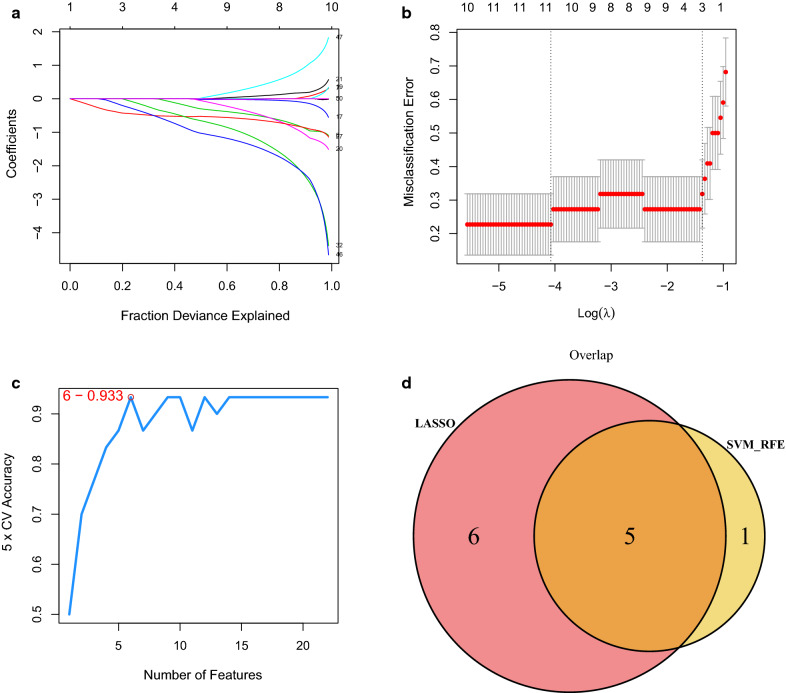
Identification of the optimal immune-related biomarkers. **a**, **b** LASSO regression analysis; **c** SVM analysis; **d** venn diagram of overlapping genes

To identify immune-related hub genes, the LASSO regression analysis for the 50 immune-related DEGs was performed to screen the gene signatures, getting 11 gene signatures (Fig. 3a, b). Besides, the SVM for the 50 immune-related DEGs was also used to screen gene signatures, getting 6 gene signatures (Fig. 3c). Subsequently, the 11 gene signatures identified by LASSO were overlapped with the 6 gene signatures identified by the SVM, and ultimately obtaining 5 hub genes [C–C motif chemokine receptor 3 (*CCR3*), major histocompatibility complex, class II, DQ alpha 1 (*HLA-DQA1*), *NCR3*, toll like receptor 3 (*TLR3*) and tumor necrosis factor (*TNF*), Fig. 3d], which were considered as the optimal immune-related biomarkers.

### Distribution of immune cells between T1DM and normal samples

**Fig. 4:**
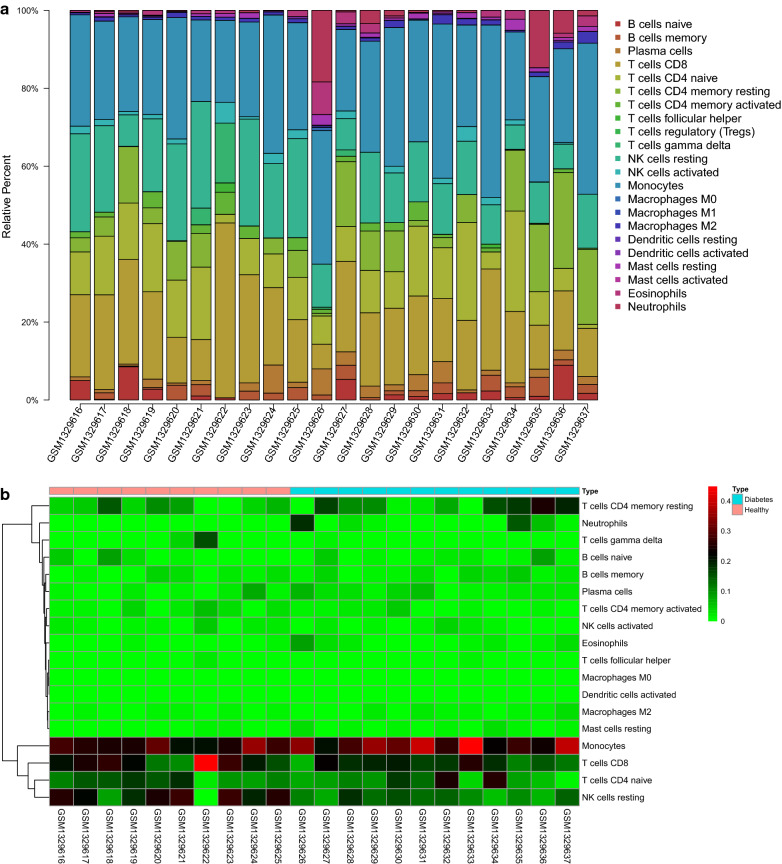

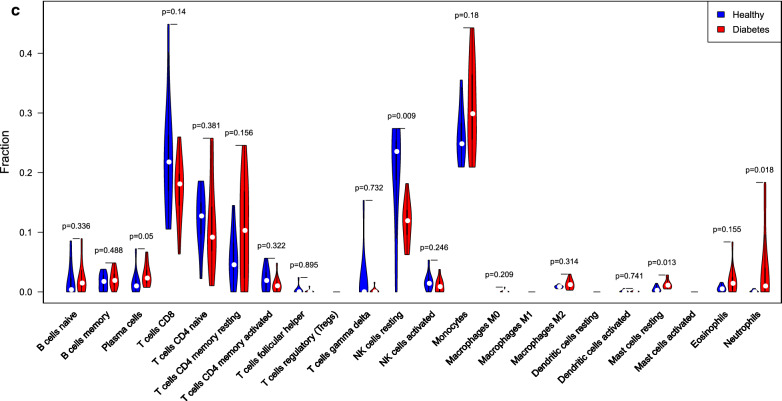
Distribution of immune cells between T1DM and normal samples. **a** Percentage of immune cells in each sample; **b** heatmap; **c** vioplot

To further investigate the correlation of the hub genes related to immune with immune cells, the distribution of the 22 immune cells between T1DM and normal samples was analyzed by the CIBERSORT algorithm. Figure 4a showed the percentage of immune cells in each sample, indicating that Monocytes, resting NK cells, resting memory CD4^+^ T cells, and CD8^+^ T cells had a larger proportion (Additional file 5: Table S5). Removing the four types of immune cells that were not in the sample, the proportion of the other immune cells in each sample was displayed by a heatmap (Fig. 4b). According to statistical results, the distribution of plasma cells (*P* = 0.05), resting mast cells (*P* = 0.013) and neutrophils (*P* = 0.018) in T1DM samples was significantly increased, while the distribution of resting NK cells (*P* = 0.009) in T1DM samples was significantly reduced, compared with that in normal samples (Fig. 4c). The above results suggested that these differential immune cells might be involved in the immune regulation process of T1DM pathogenesis.

### Correlation analysis of the immune cells and the hub genes

**Fig. 5:**
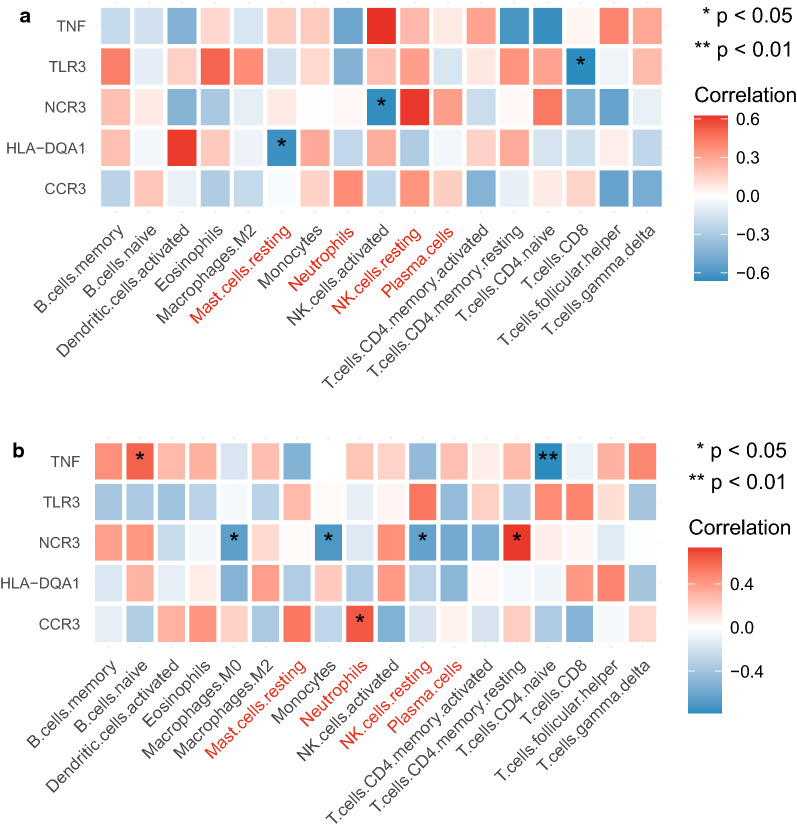
Spearman correlation of the hub genes and the immune cells. **a** normal samples; **b** T1DM samples

To further analyze the correlation of the hub genes and the immune cells, the Spearman correlation heatmaps were plotted respectively in T1DM and normal samples. As shown in Fig. 5a, b, the differential immune cells were marked with red. *NCR3* was only significantly related to the activated NK cells (*P* < 0.05) in the normal samples, while was significantly associated with multiple immune cells including M0 macrophages, monocytes, resting NK cells, and resting memory CD4^+^ T cells (all *P* < 0.05) in the T1DM samples. Moreover, *TNF* had no significant correlations to all immune cells (all *P* > 0.05) in the normal samples, but was significantly related to naive B cell (*P* < 0.05) and naive CD4^+^ T cell (*P* < 0.01) in the T1DM samples. Additionally, there were significant differences between T1DM and normal samples that *TLR3*, *HLA-DQA1* and *CCR3* were only related to CD8^+^ T cells (*P* < 0.05), resting mast cells (*P* < 0.05) and Neutrophils (*P* < 0.05), respectively. These results suggested that *NCR3* and *TNF* might play an important role in the regulation of immune cell-mediated T1DM progression.

### Construction of the hub genes and pathways network

**Fig. 6:**
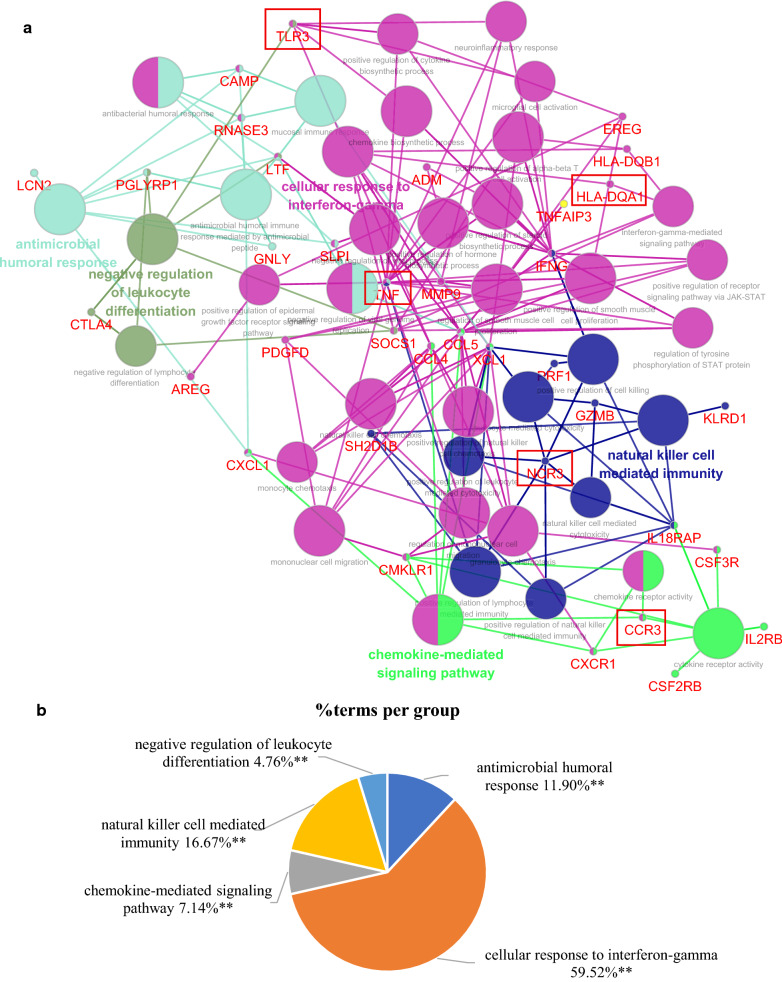

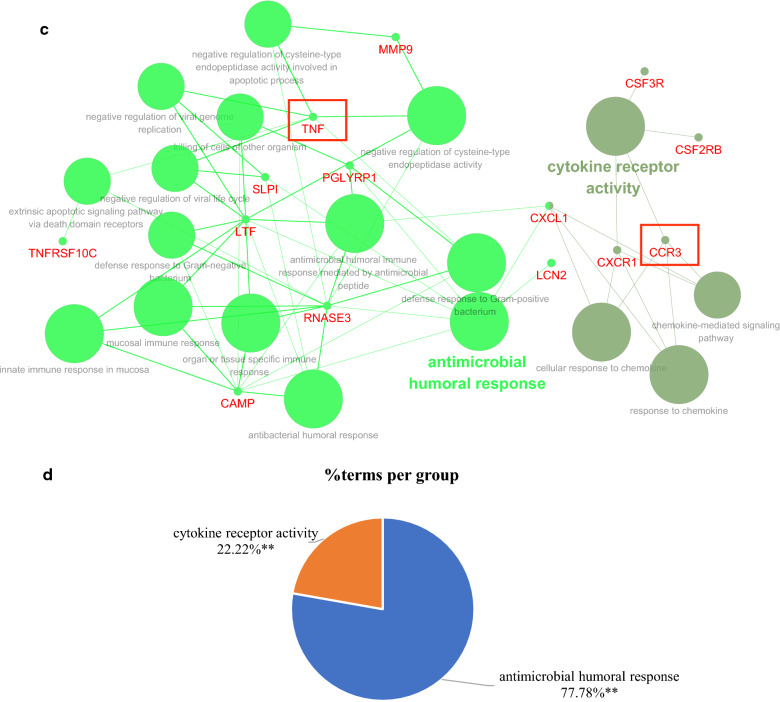

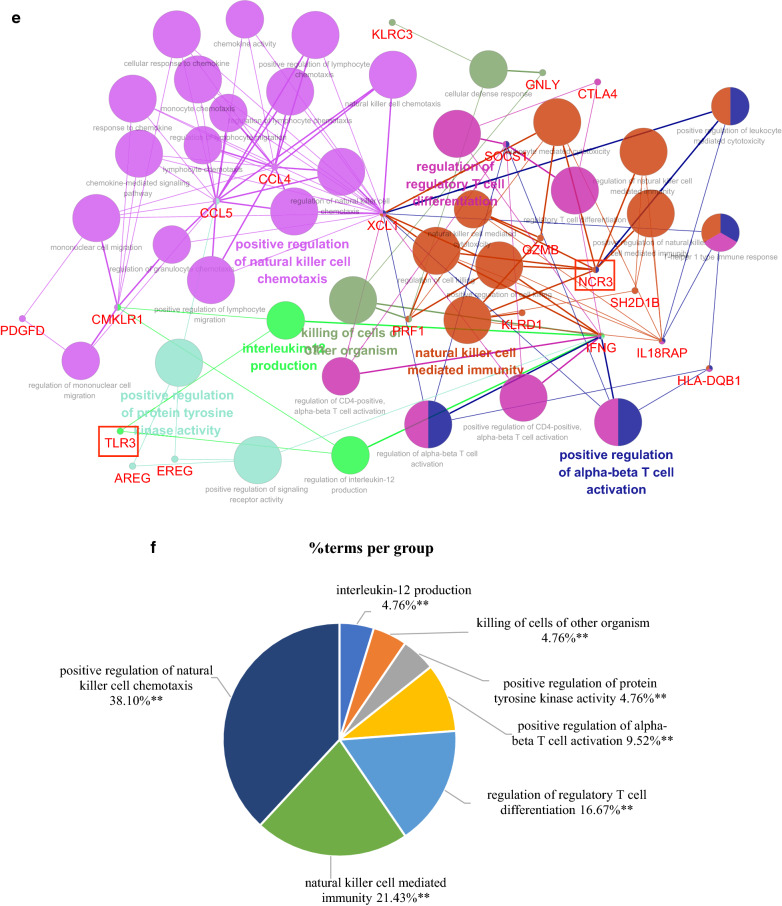
The network of hub genes and GO-BP interaction. **a** All genes;** b** the pie chart of all genes; **c** up-regulated genes; **d** the pie chart of up-regulated genes; **e** down-regulated genes; **f** the pie chart of down-regulated genes

To in-depth investigate functions of the hub genes, a network of the 50 immune-related DEGs and GO-BP interaction was constructed using ClueGO Plug-in of Cytoscape software, in which the hub genes were marked as red frame. As shown in Fig. 6a, as expected, the pathways that interacted with *TNF* were the most, mainly cellular response to interferon-gamma-related anti-infection pathways. Next was *NCR3*, which was mainly related to natural killer cell mediated immunity-related innate immune response pathways. Further, Fig. 6b also indicated that proportions of genes in cellular response to interferon-gamma and natural killer cell mediated immunity terms were the largest, 59.52% and 16.67% respectively. To more clearly show the interaction of *TNF* and *NCR3* with pathways, the up-regulated genes and down-regulated genes were separated to construct networks which were shown in Fig. 6c, d, e–f. Similar results were obtained, *TNF* participated in antimicrobial humoral response-related anti-infection pathways, *NCR3* was still involved in natural killer cell mediated immunity. Besides, the network of the 50 immune-related DEGs and KEGG interaction also indicated similar results (Additional file 6: Figure S1).

The above results suggested that *TNF* and *NCR3* might regulate T1DM progression respectively by anti-infection pathways and natural killer cell mediated immunity. NK cells recognize and kill virus-infected cells in the absence of antibodies and major histocompatibility complex (MHC), allowing for a much faster immune reaction. The role of NK cells in both the innate and adaptive immune responses is becoming increasingly important in research using NK cell activity as a potential therapy [17].

### Diagnostic value of *TNF* and *NCR3*

**Fig. 7:**
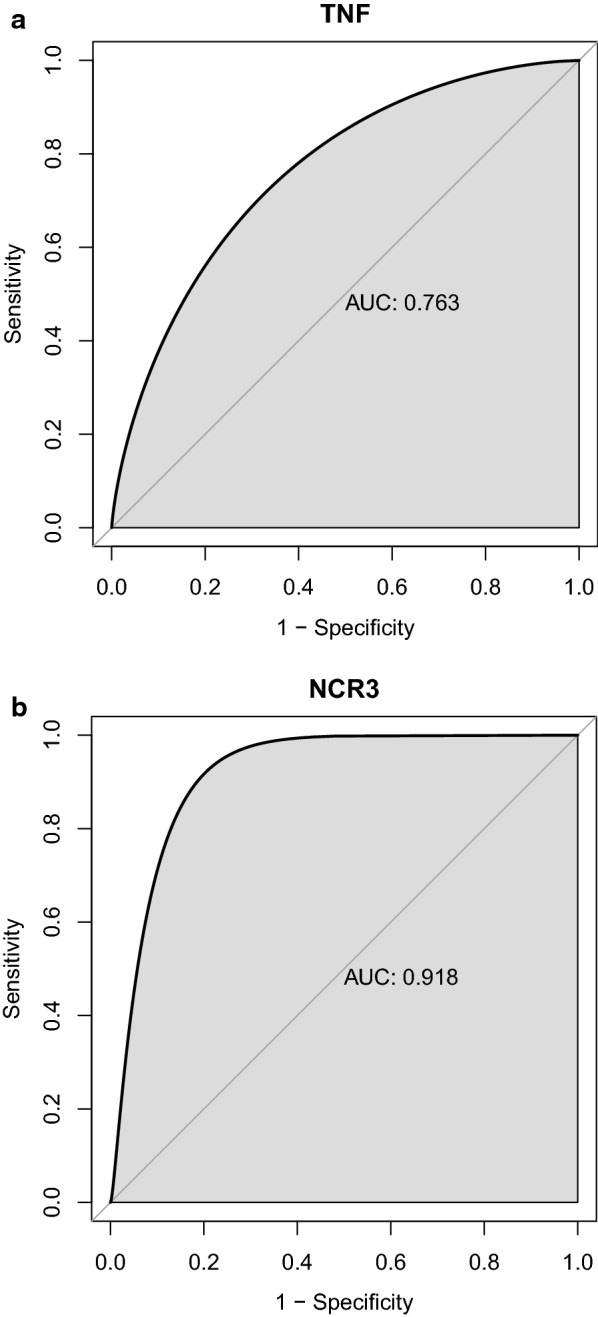
The ROC curves. **a** TNF; **b** NCR3

To explore the accuracy of the *TNF* and *NCR3* as the diagnostic biomarkers for T1DM, the ROC curves were plotted, respectively. The AUC was 0.763 (*TNF*, Fig. 7a) and 0.918 (*NCR3*, Fig. 7b), suggesting that *NCR3* possessed a higher accuracy than *TNF* in diagnosis of T1DM. And this might be related to NK cell mediated innate immune system which was often ignored in the design of novel immune-based therapies [18].

## Discussion

Immune related genes and immune cells are crucial in the pathogenesis of T1DM. However, their roles and interactions in T1DM have yet to be clarified. In this study, we identified five candidate immune-related biomarkers for T1DM and four types of immune cells differentially distributed between T1DM patients and the normal controls. Furthermore, we explored the correlations between these immune-related biomarkers and immune cells.

Firstly, we obtained 50 immune-related genes involved in T1DM by intersecting 1639 immune-related genes with 216 DEGs. Functional analysis revealed that these genes mainly participate in T1DM pathway, suggesting that T1DM is closely related to immunity. Besides, we also found some pathways other than T1DM or immunity, such as positive regulation of cytosolic calcium ion concentration and G protein-coupled receptor signaling pathway in GO enrichment analysis. Calcium ions play an important role in the maintenance of cell membrane biopotential and nerve conduction. Washburn RL et al. noted that C-peptide, a cleavage product of insulin processing unproduced by patients with T1DM, can elevate calcium concentrations by binding G protein-coupled receptors and thus treat the complications of T1DM [19]. As well, the Jak-STAT signaling pathway was present in the results of the KEGG pathway analysis. Jak-STAT signaling pathway activation can regulate the transcription of downstream genes and cell proliferation, differentiation and apoptosis process. Gurzov et al. [20] observed that the highly conserved and efficient Jak-STAT signaling pathway is necessary for normal homeostasis, and when dysregulated, it leads to the development of obesity and diabetes.

To get more robust immune-related biomarkers in T1DM, we performed LASSO and SVM analyses and found five candidate immune-related biomarkers, including CCR3, HLA-DQA1, NCR3, TLR3 and TNF. *CCR3* encodes a receptor for C–C type chemokines which belongs to family 1 of the G protein-coupled receptors [21]. Chemokine receptors play an important role in the extracellular infiltration of inflammatory factors into the inflamed tissue in T1DM insulitis [22]. According to the study of Lohmann et al. [23], there was no difference for the Th2-associated chemokine receptor *CCR3* in CD3^+^ lymphocytes between newly diagnosed T1DM patients, long-existing T1DM patients and healthy individuals. Likewise, another study also showed that there was no significant difference in the percentages of *CCR3* in recently activated circulating T cells (CD3^+^, HLA-DR^+^) between newly diagnosed T1DM patients, long standing T1DM patients and health individuals [24]. In this paper, CCR3 was differentially expressed in T1DM and normal samples, probably due to the different cell types examined in this study compared to the above studies. Thus, CCR3 may indirectly regulate the pathological process of T1DM by some mechanisms which require further research. HLA-DQA1 belongs to the HLA class II α chain paralogues. The higher expression of *HLA-DQA1* in T1DM than in normal samples affected the encoding level of DQ2.5 and DQ8 molecules on the APC surface, which can promote the presentation of self-antigens to induce autoimmunity, and present gluten antigens to homologous CD4^+^ T cells, thereby promoting T cell activation and proliferation [25]. In our study, *HLA-DQA1* was also highly expressed in T1DM patients, suggesting that it may be involved in the pathogenesis of T1DM by inducing autoimmunity and could potentially be a therapeutic target. The protein family encoded by *NCR3* is an active receptor that conveys effective signals to NK cells to lyse harmful cells and produce inflammatory cytokines [26]. Rodacki et al. [12] reported that the expression of NCR3 is reduced in patients with long-standing T1DM, which is consistent with our results. TLR3 plays a fundamental role in pathogen recognition and innate immune activation by recognizing dsRNA derived from viral replication. In some genetically susceptible individuals, this defence system does not work properly and instead induces excessive progressive inflammation and prolonged cell death, leading to the development of T1DM [27], which may explain the lower expression of *TLR3* in T1DM patients found in our study. *TNF* encodes a multifunctional pro-inflammatory cytokine secreted basically by macrophages. Qiao et al. [28] confirmed that serum TNF level in T1DM patients significantly elevated among all age, disease duration and ethnicity groups, which is similar to our result observed in PBMCs, suggesting its important role in T1DM process.

Furthermore, we analyzed the distribution of immune cells in T1DM patients and normal ones, and found that plasma cells, resting mast cells, neutrophils and resting NK cells were differentially distributed between the T1DM and the normal controls, indicating these immune cells are more important in the occurrence and development of T1DM. For plasma cells, Isabel et al. [29] found that they were increased in the thymus of non-obese diabetic (NOD) mice compared to the control ones. In another study, plasma cells were also increased in patients with diabetic nephropathy (DN). They found a positive correlation between the number of plasma cells and serum IgG levels in DN patients, probably due to antigenic stimulation promoting the activation and differentiation of naive B cells and memory B cells towards plasma cells, leading to IgG production in DN patients [30]. Our results also showed that plasma cells were increased in the samples of T1DM patients compared to the normal ones, suggesting that it may play a role in the antigen presentation process. Mast cells are a type of innate immune cells that express MHC molecules [31]. It has been demonstrated that mast cells negatively regulate T1DM and other autoimmune related diseases [32, 33]. Our findings revealed increased resting mast cell in T1DM, which also points out from a cell subtype perspective that mast cells may negatively regulate T1DM through participating in MHC expression. Neutrophils gelatinase-associated lipocalin (NGAL) has been shown to be elevated in T1DM patients and has potential to become a biomarker for DN [34, 35]. Studies suggest that neutrophil cells are involved in T1DM pathological damage through the formation of neutrophil extracellular traps (NETs) [36, 37]. Likewise, our study also indicated that neutrophils are highly expressed in T1DM. Rodacki et al. found that recently diagnosed patients with T1DM may have more, or more active, circulating NK cells, whereas patients with the long-standing T1DM had reduced cellular activity of NK cells [12]. Likewise, Lorini et al. also indicated that NK cell cytotoxic activity was reduced in patients with long-standing T1DM [38]. The reduced activity of NK cells in patients with long-standing T1DM suggests that the reduced activity of NK cells is a consequence of the disease rather than a cause. In our study, we showed that NK cell resting was less prevalent in T1DM from a cell subtype perspective, which may be due to the fact that samples in our study were from newly diagnosed T1DM patients rather than long-term T1DM patients.

In consideration that immunity requires the coordinated efforts of immune related genes and immune cells, we analyzed the correlations between five T1DM biomarkers and immune cells. We found a significantly positive correlation between TNF and naive B cells and a significantly negative correlation between TNF and naive CD4^+^ T cells in T1DM samples. Lee et al. reported that TNF promotes the proliferation of naive CD4^+^ T cells in NOD mice. It is probably due to the differences of the sources and treatment of the samples [39]. NCR3 was only negatively associated with activated NK cells in normal samples, while positively related to resting memory CD4^+^ T cells and negatively correlated with M0 macrophages, monocytes and resting NK cells in T1DM samples. It has been reported that NCR3 positively regulates NK cell activity in T1DM [12], which may explain the negative correlation between NCR3 and resting NK cells in T1DM observed in our study. Ward et al. [40] showed that a few HIV-infected CD4^+^ T cells expressed NCR3 ligands, leading to lysis of CD4^+^ T cells by NK cells. In our study, NCR3 and resting memory CD4^+^ T cells were positively correlated in patients with T1DM, by a potential mechanism that may be similar to the above study. The results of this study regarding the negative correlation of NCR3 with M0 macrophages and monocytes in T1DM patients may be a novel finding. Taken together, TNF may affect T1DM by regulating naive B cells and naive CD4^+^ T cells, while NCR3, an NK cell receptor, may affect T1DM primarily by delivering effective signals to NK cells and enable them to lyse target cells.

Interestingly, in the network of hub genes and GO-BP, we found most biological processes were mostly interacted with TNF and NCR3, and it is also a sign that they play an essential role in T1DM. Also, the network revealed the possible mechanisms of TNF and NCR3 in regulating T1DM, by anti-infection pathways and natural killer cell mediated immunity, respectively. Finally, given the importance of the above two genes, we assessed their diagnostic value by ROC and found a strong accuracy for the NCR3 (AUC = 0.918), which also provides a new biomarker for the diagnosis of T1DM.

Although we used multiple analyses to systematically investigate immune related genes, immune cells and their relationships in T1DM, this study still has some limitations due to the lack of experimental validation, single dataset and incomplete database information. Further optimization and fundamental experiments are required to reveal the detailed molecular mechanisms of these immune related genes in T1DM.

## Conclusion

In this study, we identified CCR3, HLA-DQA1, NCR3, TLR3 and TNF as potential immune-related biomarkers in T1DM. For the first time, the current study revealed the associations between immune related genes and immune cells. Our findings improve the understanding of molecular mechanisms involved in T1DM and provide novel information of diagnose and therapy for T1DM patients.

## Supplementary Information

**Additional file 1.** The 92 up-regulated genes

**Additional file 2.** The 124 down-regulated genes

**Additional file 3.** GO terms

**Additional file 4.** KEGG pathway

**Additional file 5.** Distribution of immune cells

**Additional file 6.** The network of hub genes and KEGG interaction

## Data Availability

The datasets supporting the conclusions of this article are available in the Gene Expression Omnibus (GEO) database and the ImmPort database (Immunology Database and Analysis Portal database).

## Abbreviations

AUC: Area under curve
BP: Biological processes
CC: Cellular components
CCR3: C–C motif chemokine receptor 3
DEG: Differentially expressed genes
DM: Diabetes mellitus
DN: Diabetic nephropathy
FC: Fold change
GEO: Gene Expression Omnibus
HLA-DQA1: Major histocompatibility complex, class II, DQ alpha 1
IDF: International Diabetes Federation
ImmPort database: Immunology Database and Analysis Portal database
LASSO: Least absolute shrinkage and selection operator
MF: Molecular functions
NCBI: National Center of Biotechnology Information
NCR3: Natural cytotoxicity triggering receptor 3
NETs: Neutrophil extracellular traps
NGAL: Neutrophils gelatinase-associated lipocalin
NOD: Non-obese diabetic
PBMCs: Peripheral blood mononuclear cells
ROC: Receiver operating characteristic
SVM: Support vector machines
T1DM: Type 1 diabetes mellitus
T2DM: Type 2 diabetes mellitus
TLR3: Toll like receptor 3
TNF: Tumor necrosis factor
Tregs: Regulatory T cells
