# Supplementary figures and images for: Analysis of immune cell components and immune-related gene expression profiles in peripheral blood of patients with type 1 diabetes mellitus
Source: J Transl Med. 2021 Jul 26;19:319. doi: 10.1186/s12967-021-02991-3 (PMC8314644; doi:10.1186/s12967-021-02991-3)

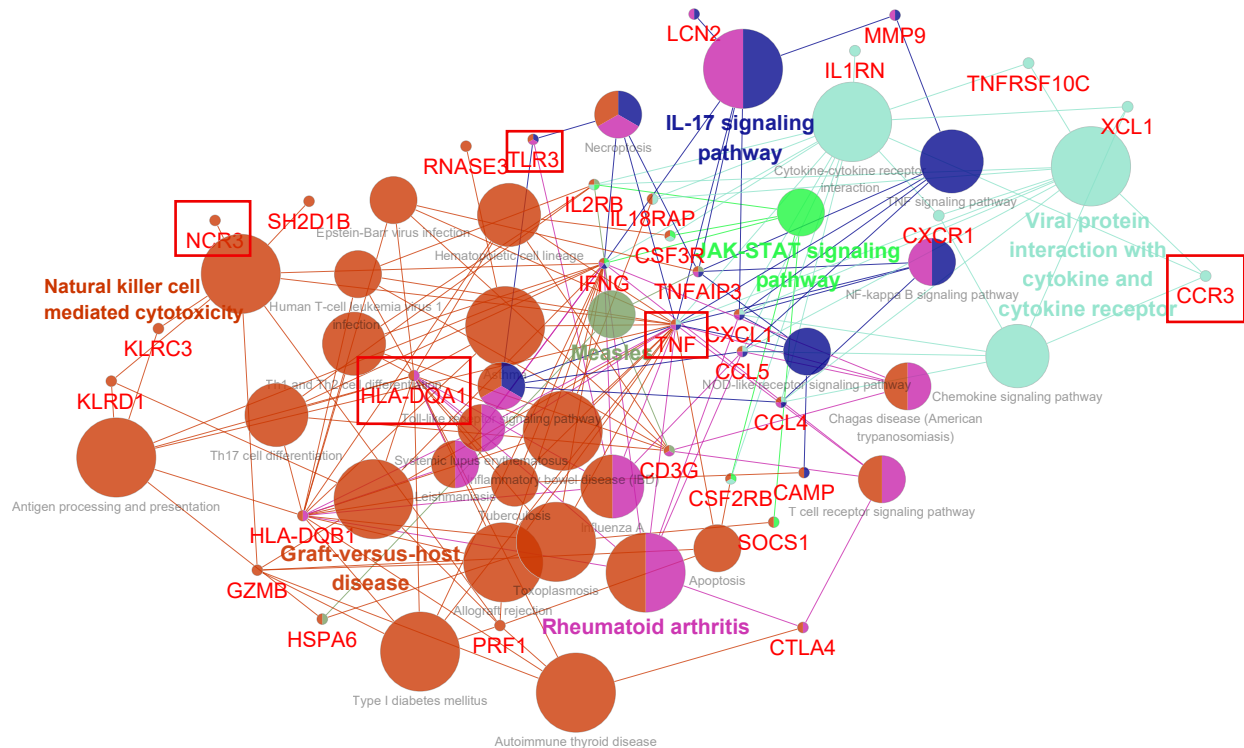

Supplement: Supplementary file 6 — Additional file 6. The network of hub genes and KEGG interaction [file 12967_2021_2991_MOESM6_ESM.zip › Figure S1a-all_KEGG.pdf]

## %terms per group

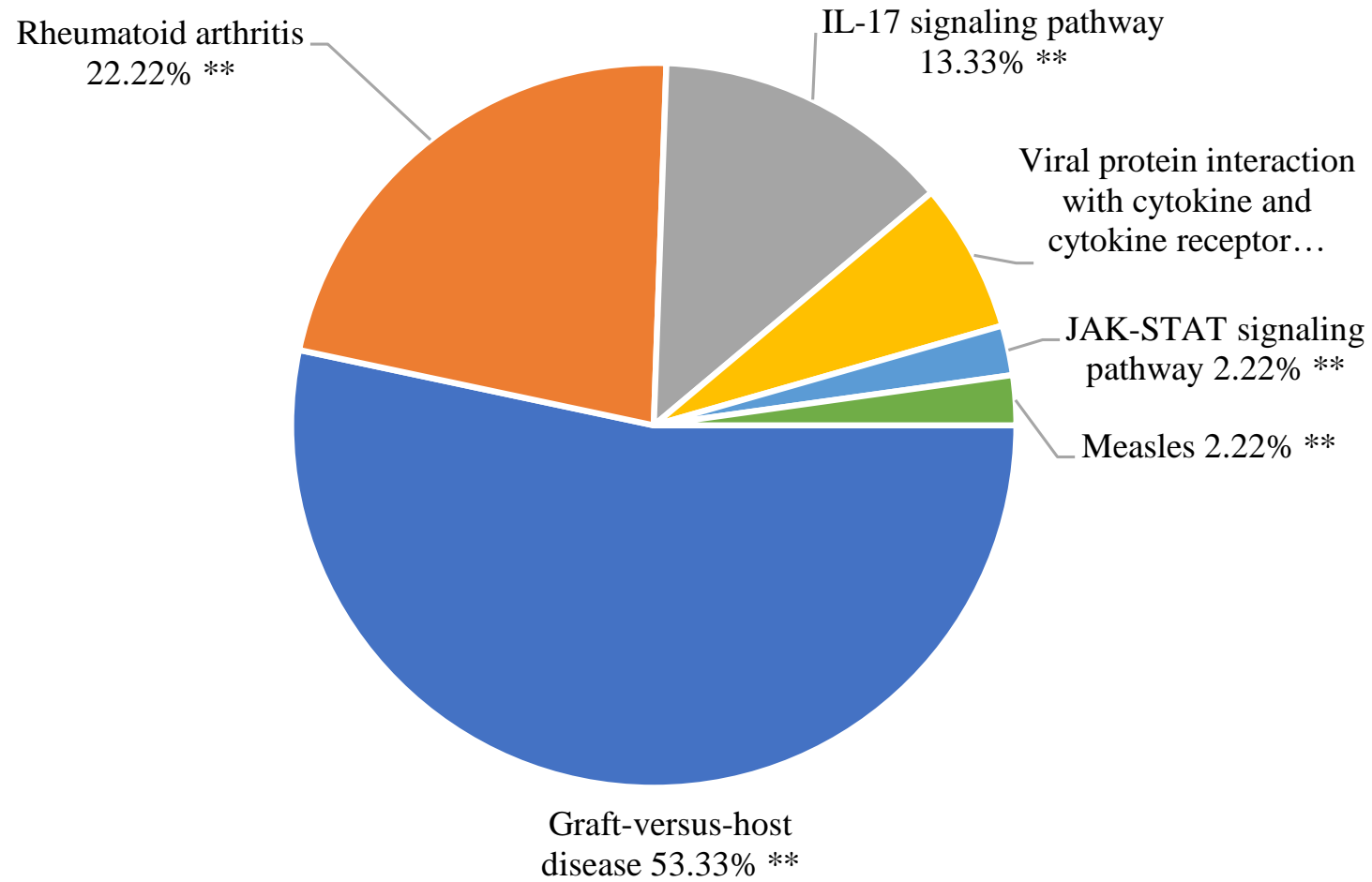

Supplement: Supplementary file 6 — Additional file 6. The network of hub genes and KEGG interaction [file 12967_2021_2991_MOESM6_ESM.zip › Figure S1b-all_KEGG.pdf]

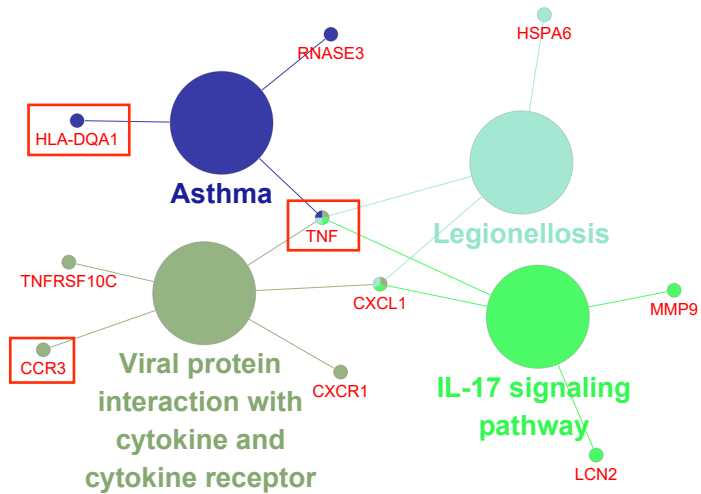

Supplement: Supplementary file 6 — Additional file 6. The network of hub genes and KEGG interaction [file 12967_2021_2991_MOESM6_ESM.zip › Figure S1c-KEGG_Up.pdf]

## %terms per group

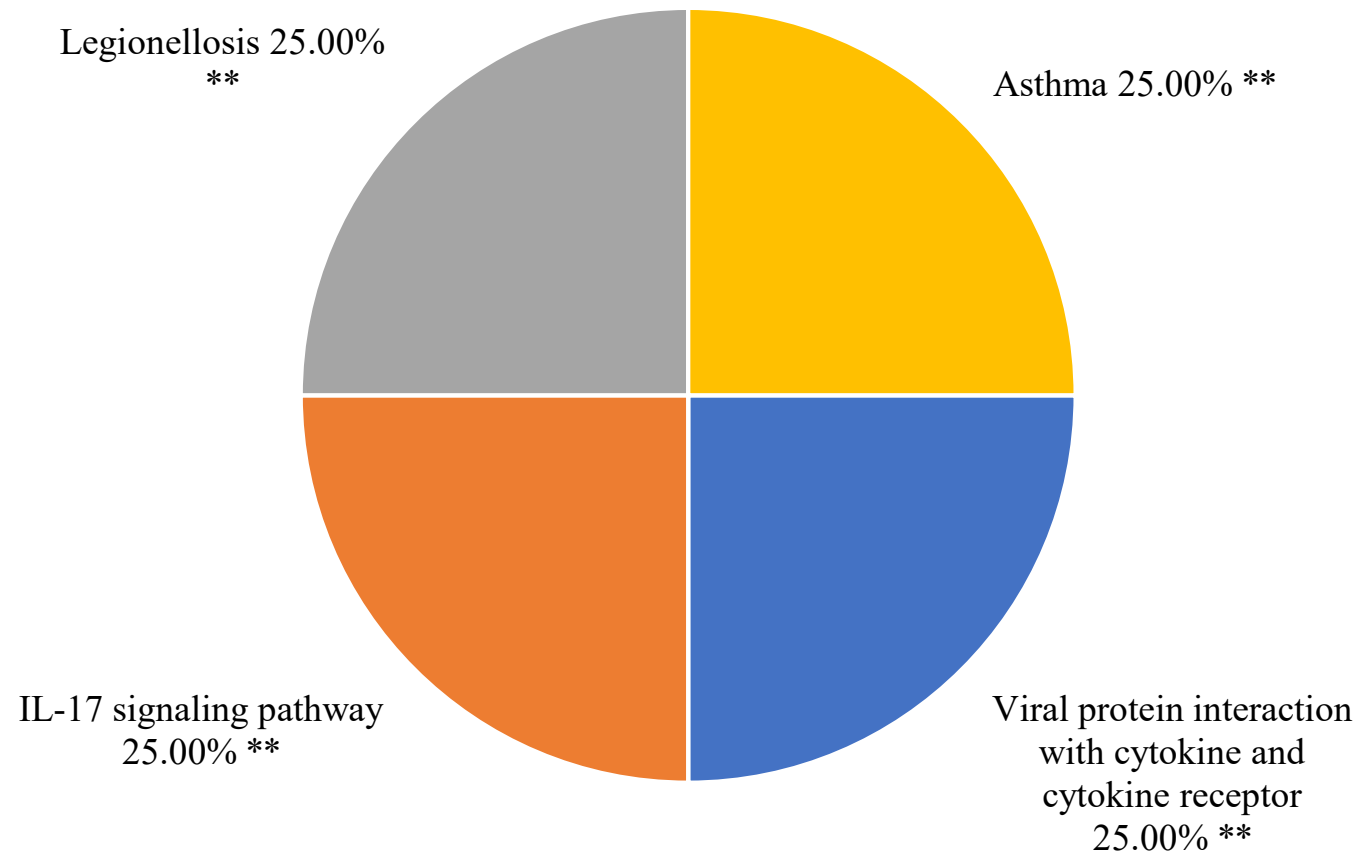

Supplement: Supplementary file 6 — Additional file 6. The network of hub genes and KEGG interaction [file 12967_2021_2991_MOESM6_ESM.zip › Figure S1d-KEGG-Up.pdf]

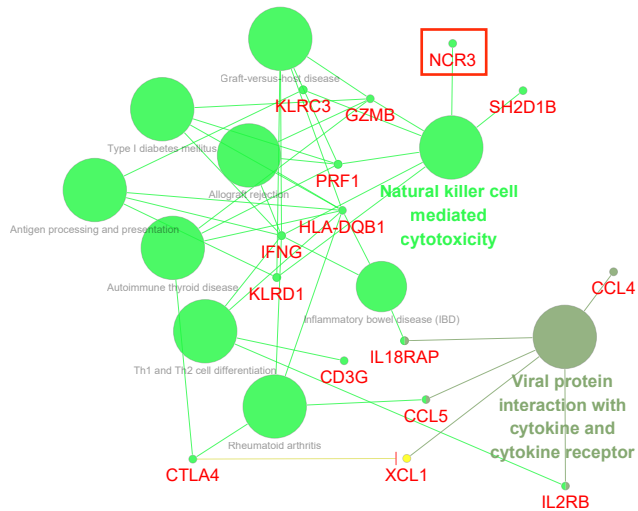

Supplement: Supplementary file 6 — Additional file 6. The network of hub genes and KEGG interaction [file 12967_2021_2991_MOESM6_ESM.zip › Figure S1e-KEGG_Down.pdf]

## %terms per group

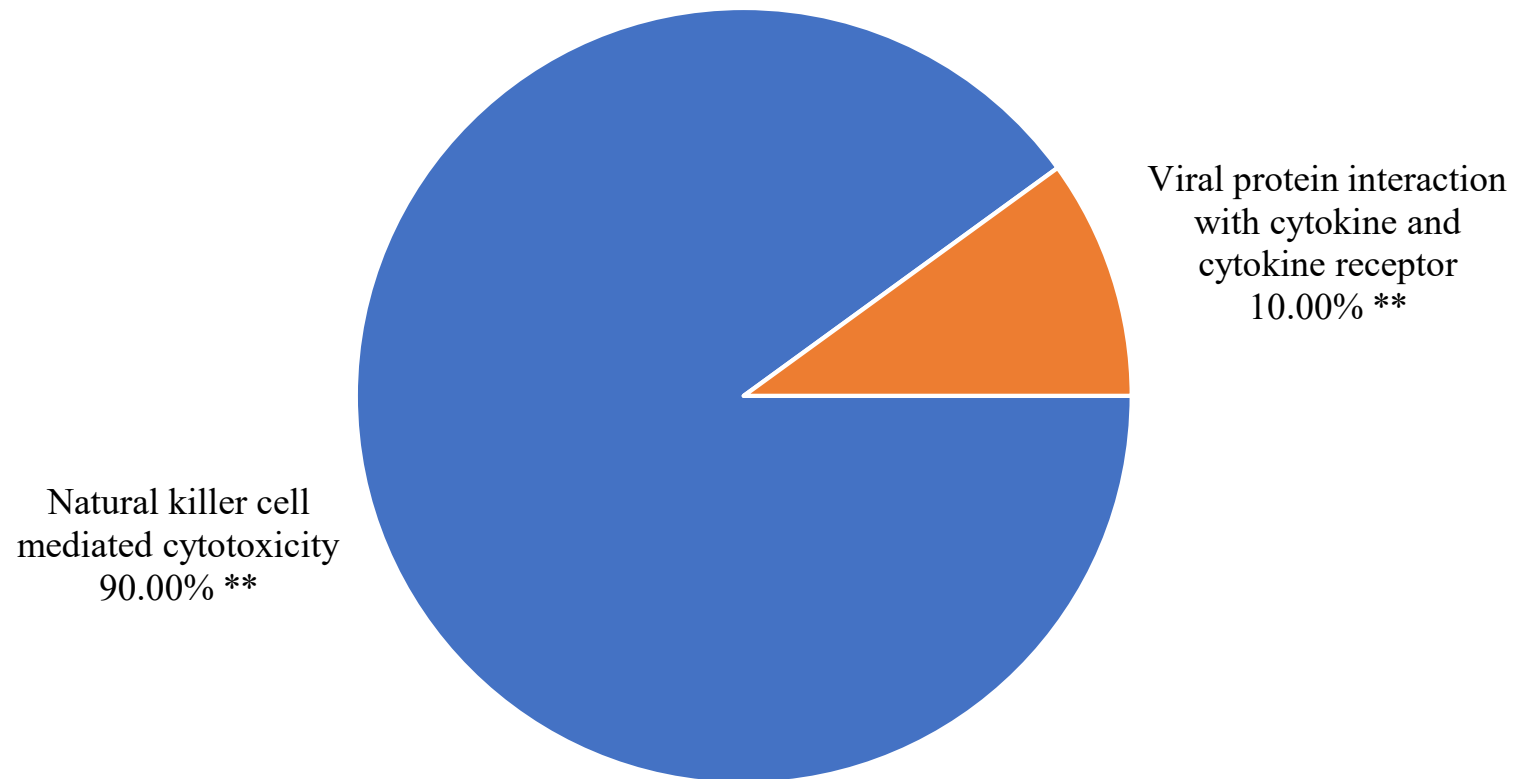

Supplement: Supplementary file 6 — Additional file 6. The network of hub genes and KEGG interaction [file 12967_2021_2991_MOESM6_ESM.zip › Figure S1f-KEGG-Down.pdf]
